# Identification of Volatile Organic Compounds as Natural Antifungal Agents Against *Botrytis cinerea* in Grape-Based Systems

**DOI:** 10.3390/foods15010119

**Published:** 2026-01-01

**Authors:** Mitja Martelanc, Tatjana Radovanović Vukajlović, Melita Sternad Lemut, Lenart Žežlina, Lorena Butinar

**Affiliations:** 1 Wine Research Centre, University of Nova Gorica, Glavni trg 8, SI-5271 Vipava, Slovenia; mitja.martelanc@ung.si (M.M.); tatjana.radovanovic@ung.si (T.R.V.); msternadlemut@ung.si (M.S.L.); 2 Biotechnical Faculty, University of Ljubljana, Jamnikarjeva Ulica 101, SI-1000 Ljubljana, Slovenia; lenart.zezlina@gmail.com

**Keywords:** volatile organic compounds, grape, HS-SPME-GC-MS, validation, biocontrol, yeast, *Botrytis cinerea*

## Abstract

*Botrytis cinerea* Pers., the causal agent of grey mould, causes major economic losses in viticulture by reducing grape and wine quality and yield. Antagonistic yeasts that release bioactive volatile organic compounds (VOCs) represent a sustainable alternative to synthetic fungicides. Here, VOCs produced by *Pichia guilliermondii* strain ZIM624 were identified and assessed for antifungal activity against *B. cinerea*. 65 VOCs—including higher alcohols, volatile phenols, esters, and terpenes—were detected using two newly developed and validated analytical methods combining automated headspace solid-phase microextraction with gas chromatography–mass spectrometry. A total of 13 VOCs were selected for the bioassays. Fumigation assays demonstrated that terpenes (citronellol, geraniol, nerol, α-terpineol, and linalool) were the most effective inhibitors of *B. cinerea* mycelial growth (EC_50_ = 6.3–33.9 μL/L). Strong inhibition was also observed for 4-vinylphenol and isoamyl acetate. In vivo assays confirmed that exposing infected grape berries to *P. guilliermondii* VOCs significantly reduced grey mould incidence. These results highlight the potential of *P. guilliermondii* ZIM624 volatiles as natural biofumigants for the eco-friendly management of *B. cinerea* in grapes. Future research should focus on optimising VOC production, evaluating efficacy under field conditions, and developing formulations for practical application in vineyards and post-harvest storage. Additionally, investigating potential synergistic effects of VOC combinations could lead to more effective biocontrol strategies.

## 1. Introduction

The ubiquitous filamentous fungus *Botrytis cinerea* is among the most destructive plant pathogens, infecting more than 200 host species worldwide [1,2]. In viticulture, it is the causal agent of grey mould, a disease responsible for major yield losses and severe reductions in grape and wine quality. Current management practices rely heavily on synthetic fungicides [3]; however, their use is increasingly restricted because of adverse effects on human health, environmental concerns, and the emergence of fungicide-resistant pathogen strains [4]. Consequently, research efforts are shifting toward environmentally sustainable alternatives, particularly natural products, such as elicitors of host defense responses and microbial antagonists [5,6,7].

Antagonistic yeasts have emerged as promising biocontrol agents against *B. cinerea* [5,8,9]. More than 40 yeast species that are naturally associated with grapes and wine [10] have been reported to exhibit antifungal activity [8,9,11]. Recent work has also shown that newly selected non-*Saccharomyces* yeasts can act as effective biocontrol agents in table grapes, demonstrating their technological potential for managing *B. cinerea* infections [12]. Compared to filamentous fungi, yeasts offer several advantages: they do not produce mycotoxins or allergenic spores, can be mass-produced on inexpensive substrates, and readily adapt to vineyard and grape microenvironments [13,14]. Their antagonistic mechanisms include competition for nutrients and space [15], secretion of lytic enzymes such as chitinases and glucanases [16,17], biofilm formation [18,19], and, importantly, the production of antifungal metabolites such as volatile organic compounds (VOCs) [20].

VOCs are small, low molecular weight compounds (<300 Da) with high vapour pressure and low water solubility [21]. They comprise diverse chemical classes, including hydrocarbons, alcohols, aldehydes, ketones, esters, terpenes, and phenols [22]. Several yeast species, including *Wickerhamomyces anomalus*, *Metschnikowia pulcherrima*, *Saccharomyces cerevisiae*, and *Aureobasidium pullulans*, have been shown to inhibit *B. cinerea* in vitro through VOC release [15]. Specific antifungal VOCs have been identified, such as 2-ethyl-1-hexanol from *Sporidiobolus pararoseus* [23], and 1,3,5,7-cyclooctatetraene, 3-methyl-1-butanol, 2-nonanone, and phenylethyl alcohol from *Candida intermedia* [20].

Different classes of VOCs have distinct origins. Esters, which are absent in grapes but formed during fermentation, arise through yeast enzymes encoded by the genes *ATF1*/*ATF2* and *EHT1*/*EEB1*, producing acetate and ethyl esters that impart banana, pear, apple, and pineapple notes [24,25,26]. In contrast, terpenes are grape-derived and occur free or as glycosides. Hydrolysis during winemaking releases monoterpenes, such as linalool, geraniol, and citronellol, which are responsible for floral and citrus aromas, whereas yeasts mainly aid their release [26]. C6 alcohols (“leaf alcohols”) originate from fatty acid oxidation in the damaged berries. Aldehydes, such as hexanal, provide grassy notes but are reduced to alcohols (e.g., hexanol, cis-3-hexenol) during fermentation, with levels depending on grape handling and oxygen exposure [27]. Nor-isoprenoids are derived from carotenoid degradation and are usually released from glycosidic precursors during fermentation or aging. Key examples include β-damascenone (floral, fruity) and TDN (kerosene in aged Riesling), which are influenced by grape variety, ripeness, and sunlight [28].

In grapevines, these secondary metabolites—including esters, terpenes, C6 alcohols, and nor-isoprenoids—not only determine grape and wine aroma but also fulfil ecological functions. They can inhibit microbial pathogens, deter herbivores, and attract natural enemies of pests [29]. Some volatiles act as signalling molecules, mediating plant–plant communication and enhancing systemic resistance in neighbouring vines [30]. In addition, grape-associated yeasts contribute further volatile metabolites during fermentation, including esters, higher alcohols, and terpenes, often at low yet biologically active concentrations.

Given the structural diversity and wide polarity range of VOCs, selective and quantitative extraction is a prerequisite for their reliable analysis. Among the available methods, headspace solid-phase microextraction (HS-SPME) coupled with gas chromatography–mass spectrometry (GC–MS) has become the preferred approach, offering solvent-free operation, high sensitivity, and compatibility with automation [31,32,33,34]. Although yeast-modulated VOCs have been qualitatively characterised in co-cultures with fungi [20,35,36], quantitative assessments of these compounds in grape-based matrices remain scarce. To our knowledge, this is the first study to combine the validated quantification of yeast-modulated VOCs in multiple grape-based media and in vivo bioassays of selected synthetic VOCs compared across chemical classes and yeast VOCs in grape berries.

To address these gaps, the present study aimed to (i) develop and validate two robust HS-SPME–GC–MS methods for quantifying volatile phenols, esters, higher alcohols, and terpenes produced by biocontrol yeast *Pichia guilliermondii* ZIM624 in grape-based media; and (ii) identify yeast-derived VOCs with antifungal activity against *B. cinerea in vitro* and *in vivo* on grape berries.

## 2. Materials and Methods

### 2.1. Reagents, Materials and Standards for HS-SPME-GC-MS Analyses

Esters: Standards of propyl acetate (99%), isobutyl acetate (97%), ethyl 2-methylbutyrate (99%), phenylethyl acetate (98%), trans-2-hexen-1-ol (96%), cis-3-hexen-1-ol (98%), trans-3-hexen-1-ol (97%), ethyl propanoate (97%), butyl acetate (99%), hexanol (99%), Z-3-hexenyl acetate (98%), E-2-hexenyl acetate (98%), and ethyl dihydrocinnamate were obtained from Sigma-Aldrich (Steinheim, Germany). Ethyl leucate (98%) was purchased from Tokyo Chemical Industry (Tokyo, Japan). Ethyl butyrate (99%), hexyl acetate (99%), isoamyl acetate (99%), ethyl isovalerate (99%), ethyl decanoate (99%), octyl acetate (99%), ethyl hexanoate (99%), ethyl octanoate (99%), and ethylphenyl acetate (99%) were obtained from Acros Organics (Geel, Belgium). Ethyl isobutyrate (98%) and ethyl cinnamate (98%) were purchased from Alfa Aesar (Karlsruhe, Germany).

Internal standards for the esters: Ethyl butyrate-4,4,4-d_3_ (99.8%), ethyl hexanoate-d_11_ (98.7%), ethyl octanoate-d_15_ (98.8%), and ethyl trans-cinnamate-d_5_ (99.4%) were obtained from C/D/N Isotopes Inc. (Pointe-Claire, QC, Canada).

Volatile phenols: Methyl salicylate (99%), 4-ethylguaiacol (98%), guaiacol (99%), and 2-methoxy-4-vinylphenol (4-vinylguaiacol, 98%) were purchased from Sigma-Aldrich (Steinheim, Germany). 4-Vinylphenol (10%) was obtained from BIOSYNTH Carbosynth (Compton, UK) and 4-ethylphenol (97%) from Acros Organics.

Terpenes and norisoprenoids. Standards of 3-carene (95%), α-terpinene (95%), γ-terpinene (97%), 1,4-cineole (95%), eucalyptol (99%), p-cymene (99.5%), α-terpinolene (95%), α-ionone (90%), β-ionone (96%), linalool (97%), cis-geraniol (97%), rose oxide (99%), 4-terpineol (95%), trans-β-damascenone (98%), nerolidol (97%), and linalool oxide (cis and trans, 97%) were purchased from Sigma-Aldrich (Steinheim, Germany). α-Terpineol (97%) was obtained from Thermo Scientific (Acros Organics, Morris Plains, NJ, USA). Citronellol (95%), trans-geraniol (99%), and limonene (96%) were purchased from Acros Organics. TDN (1,1,6-trimethyl-1,2-dihydronaphthalene, 80%) was obtained from BIOSYNTH Carbosynth (Compton, UK). Vitispirane 1 and 2 (98%) and hotrienol (97%) were obtained from Eptes Analytical (Vevey, Switzerland).

Internal standards for the terpenes and norisoprenoids: Geraniol-d_6_, linalool-d_5_, 2-octanol, and ethyl trans-cinnamate-d_5_ were used as internal standards. Deuterated compounds were purchased from Eptes Sarl (Vevey, Switzerland).

Solvents and reagents: Absolute ethanol (99.8%, HPLC grade) was obtained from Honeywell (Riedel-de Haën, Seelze, Germany). Sodium chloride (≥99% purity) was supplied by Honeywell (Fluka, Seelze, Germany). Ultrapure water was prepared using a Milli-Q water purification system (Purelab Option-Q, ELGA LabWater, High Wycombe, Buckinghamshire, UK), yielding a resistivity of >18.0 MΩ·cm^−1^ at 25 °C.

### 2.2. VOCs Production by P. guilliermondii ZIM624 in Diluted Grape Juice and Artificial Grape Medium

In this study, we focused on identifying VOCs produced by the biocontrol yeast *Pichia guilliermondii* ZIM624 (Zbirka industrijskih mikroorganizmov, ZIM Culture Collection, Ljubljana, Slovenia), which was selected based on previous reports of its antifungal mechanisms [37,38,39].

Yeast was cultured in two grape-based media: diluted Pinot noir grape (*Vitis vinifera* L. cv. Pinot noir) juice medium (GJM) [40] and synthetic complete medium (SCM; 0.17% (*w*/*v*) Difco™ YNB without amino acids, 0.5% (*w*/*v*) ammonium sulphate, 2% (*w*/*v*) glucose) [41] supplemented with 2% (*w*/*v*) dried Pinot noir grape skins.

A yeast pre-culture was prepared by inoculating a loopful of a 2-day-old colony from yeast peptone dextrose (YPD) agar (1% (*w*/*v*) yeast extract, 2% (*w*/*v*) peptone, 2% (*w*/*v*) glucose, and 2% (*w*/*v*) agar) into 25 mL of YPD broth, followed by incubation at 25 °C with shaking at 150 rpm. The overnight culture was centrifuged (4000 rpm, 10 min, room temperature), washed, and resuspended in 0.85% (*w*/*v*) NaCl to a final concentration of 1.15 × 10^8^ cells/mL.

For VOC production, 100 μL of the prepared yeast suspension was inoculated into 25 mL of GJM in 40 mL glass vials (in triplicate) and incubated at 25 °C, either under static conditions or with agitation at 150 rpm. Identical conditions were applied to cultures prepared in SCM supplemented with dried Pinot noir grape skins. After 3 days of incubation at 25 °C, the cultures were centrifuged (2000 rpm, 15 min, 20 °C), and the supernatants were collected and stored in glass vials at −20 °C until further analysis of VOCs by HS-SPME–GC–MS.

### 2.3. Preparation of Standard Solutions

The analysed compounds were identified by comparing their retention times and mass spectra with those of authentic standards. The identities were further confirmed using the NIST mass spectral library.

For quantification, internal standards were spiked into the samples as described by Antalick et al. [31] with modifications. Each internal standard was first dissolved in ethanol to prepare the individual stock solutions. Two mixed stock solutions were then prepared: one containing terpenes and the other containing esters and volatile phenols. The concentration levels of all solutions are presented in Table S1. Stock and mixed solutions were stored at −80 °C until use.

### 2.4. Sample Preparation and HS-SPME-GC-MS Analysis

For sample preparation, 1 mL of the culture supernatant was transferred into a 20 mL SPME vial containing 2 g NaCl and 9 mL deionised water. The samples were spiked with 20 μL of the internal standard solution, sealed with a PTFE-lined cap, and kept at 10 °C in a cooled autosampler tray (Peltier system).

Each sample was homogenised at 250 rpm for 10 min using an Agitator Module on a Gerstel MPS Robotic autosampler. VOCs were extracted by HS-SPME using a fibre assembly (50/30 μm DVB/CAR/PDMS, Stableflex, 24 Ga; Supelco, Bellefonte, Centre County, PA, USA). The fibre was conditioned for 2 min at 270 °C and then exposed to the vial headspace for 30 min at 40 °C with continuous agitation (250 rpm). Desorption was performed in the GC injector at 250 °C for 15 min with an injection time of 30 s. Fibres were cleaned between runs for 10 min at 270 °C.

Gas chromatographic analyses were performed using an HP 8890 GC system coupled to a 5977B GC/MSD quadrupole mass spectrometer equipped with a Gerstel MPS Robotic autosampler. The injections were splitless (0.5 min) using an ultra-inert single-taper liner. Separation was achieved on a DB-WAX UI capillary column (60 m × 0.25 mm × 0.25 μm, Agilent Technologies, Santa Clara, CA, USA) with helium (He 6.0) as the carrier gas at 1.5 mL/min. The oven programme for esters, C6 alcohols, and volatile phenols was as follows: 40 °C (5 min), increased to 200 °C at 3 °C/min, then to 240 °C at 10 °C/min, and held for 10 min. The oven programme for terpenes and norisoprenoids was as follows: 40 °C (5 min), increased to 65 °C at 3 °C/min (10 min hold), then to 90 °C at 3 °C/min, to 130 °C at 10 °C/min, and finally to 240 °C at 4 °C/min (10 min hold).

The MS was operated in electron ionisation mode (70 eV) in the selected-ion monitoring (SIM) mode. The source and quadrupole temperatures were set at 230 °C and 150 °C, respectively. The total runtime was 72.3 min. The monitored ions for all compounds and internal standards are listed in Supplementary Tables S1 and S2. Data acquisition and processing were performed using the MassHunter software (version F.01.03 SR1; Agilent Technologies, Santa Clara, CA, USA).

### 2.5. Standard and Quality Control Samples

Standard and internal standard stock solutions were prepared in ethanol. Working solutions were prepared by dilution in ethanol, including separate solutions for the deuterated internal standards. All solutions were stored in amber vials at −20 °C.

The calibration curves consisted of seven points. The highest calibration point was prepared by spiking an appropriate volume of the working solution into 100 mL of the medium, and the remaining points were obtained by serial dilution. Recovery test solutions (midpoint of the calibration curve) and control samples were prepared in the same manner.

### 2.6. Method Validation

Method validation was performed following the Eurachem Guide: The Fitness for Purpose of Analytical Methods [42]. Both HS-SPME–GC–MS methods were validated using grape juice medium (GJM) and synthetic medium with dried grape skins (SCM). The validation parameters included linearity, accuracy (recovery), repeatability, inter-day precision, carry-over, limit of detection (LOD, S/N = 3), limit of quantification (LOQ, S/N = 10), and quality control (QC) performance. Detailed numerical data and calibration model parameters (slope and intercept with standard errors) are provided in Tables S2 and S3 (Supplementary Materials). Separate calibration curves were established for the GJM and SCM to account for matrix differences and precursor availability, which can influence the extraction efficiency and analyte response. This matrix-specific calibration is widely used and minimises the bias that would result from applying a single calibration across both matrices. Each analyte was paired with an appropriate internal standard (IS) based on its structural and chromatographic similarities. The single-ion monitoring (SIM) ions used for quantification and confirmation, along with internal standard associations, are summarised in Table S2 (Supplementary Materials). For analytes without a directly corresponding IS, the most chemically and chromatographically related standard was applied to correct for matrix effects. Recovery and repeatability were assessed using samples spiked at the calibration midpoint. Repeatability was evaluated through six consecutive injections of a single spiked sample, while inter-day precision was assessed using three replicates per day over five consecutive days. Given that each analytical run lasted approximately 72 min, this approach effectively captured both intra-day and inter-day variability. Recovery at the midpoint was considered sufficient because the study focused on relative changes in analyte concentrations before and after yeast activity, rather than absolute quantification. Carry-over was below 1% for all analytes, and QC samples were included in each batch, along with two blanks, to monitor contamination. QC trends across the 14-sample sequences showed no significant drift within the repeatability deviations. Samples were maintained at 10 °C in the autosampler, and based on literature-reported stability [43,44] and our QC results, no significant analyte loss occurred within the batch. LOD and LOQ were determined as signal-to-noise ratios (S/N) of 3 and 10, respectively, calculated in SIM mode by comparing the peak height of the analyte ion to the standard deviation of the background noise measured adjacent to the peak. Although specific numerical LOD and LOQ values for each analyte were not tabulated, all analytes were detected at concentrations at least three times higher than their LOQs, confirming sufficient sensitivity for the study objectives. The methods were adapted from two previously validated protocols—one for ester analysis and one for terpene analysis—which employed the same equipment, fibre type, and extraction parameters [43,44]. Given the slight differences in the sample matrices, additional validation was performed to confirm the accuracy and reliability of the adapted methods used in the current study.

### 2.7. Antifungal Activity of Selected Synthetic VOCs Against B. cinerea Growth

The antifungal activity of 13 synthetic VOCs was tested against *B. cinerea*: isoamyl acetate, isobutyl acetate, ethyl propionate, ethyl butyrate, α-terpineol, eucalyptol, citronellol, geraniol, linalool, nerol, vitispirane, 4-vinyl phenol, and 4-vinyl guaiacol. The assay was performed according to Huang et al. [20], with minor modifications. *B. cinerea* F61 (ZIM culture collection) was pre-cultured on potato dextrose agar (PDA) (Biolife, Italy) for 2 days. For each assay, 100 μL of a VOC dilution in absolute ethanol (0.1–50% *v*/*v*) was applied to a sterile filter paper strip (1.6 × 1.5 cm) placed at the centre of a 9 cm Petri dish. Ethanol alone served as the control. The same volume of 100 μL ethanol was used for the solvent control. This ethanol load corresponds to 0 µL of VOC per litre of headspace and is included as the 0 µL/L dose in the fitted dose–response curves (see below).

The dish was covered with a PDA plate inoculated with a mycelial plug (6 mm diameter) of *B. cinerea*. Three replicates were performed per compound concentration. The plates were sealed with Parafilm and incubated at room temperature for 3 days.

After incubation, colony diameters were measured, and inhibition was expressed as the concentration required for 50% growth reduction (EC_50_). EC_50_ values were calculated as µL of VOC per litre of headspace above the PDA plate, according to Strobel et al. [45]. The VOC chamber consisted of two 9 cm polystyrene Petri dishes (total internal volume 170 mL). After subtracting the 17 mL occupied by the PDA layer, the effective headspace volume was 153 mL. Dose–response relationships were fitted using the four-parameter log-logistic (LL.4) model and the constant relative slope (CRS.4c) model in the R package drc (version 3.0.1) [46].

### 2.8. In Vivo Assay of Antifungal VOCs Activity Produced by P. guilliermondii ZIM624 Against B. cinerea in Grape Berries

The antifungal activity of the VOCs produced by *P. guilliermondii* ZIM624 against *B. cinerea* F61 was evaluated using grape berries (*Vitis vinifera* L. cv. Pinot noir), collected in mid-August 2022. Berries were rinsed with sterile double-deionised water, surface-sanitised by immersion in 70% ethanol for 3 s, rinsed again with sterile water, and air-dried using sterile paper towels.

A *B. cinerea* conidial suspension was prepared from 7-day-old cultures grown on potato dextrose agar (PDA) slants. Spores were washed with spore suspension solution (SSS; 0.05% (*v*/*v*) Tween 80), filtered through sterile steel wool, centrifuged at 10,000× *g* for 10 min at room temperature, and resuspended in SSS to a final concentration of 10^6^ conidia/mL. For berry inoculation, wounds (2 mm diameter, 3 mm depth) were made with a sterile pipette tip, and 5 µL of the conidial suspension was applied to each wound.

Yeast inoculum was prepared by transferring a loop of *P. guilliermondii* ZIM624 into 25 mL of YPD broth and incubating overnight at 25 °C with shaking at 150 rpm. The following day, 100 µL of the yeast suspension (10^8^ cells/mL) was spread onto solid GJM prepared by pouring 60 mL of GJM into square Petri dishes (12 × 12 cm). After incubation at 25 °C for 24 h, the actively growing cultures were used for the assay.

Five experimental groups were established, each performed in triplicate with 20 berries per replicate: (i) *B. cinerea*-infected berries exposed to VOCs from *P. guilliermondii* ZIM624 grown on GJM; (ii) *B. cinerea*-infected berries exposed to sterile GJM (negative control); (iii) Wounded but non-infected berries exposed to *P. guilliermondii* VOCs; (iv) Sound berries exposed to *P. guilliermondii* VOCs; (v) Sound berries exposed to sterile GJM.

In each case, the berries were placed on a sterile stainless-steel net above the corresponding GJM plates, and the entire setup was enclosed in sterile plastic bags. The estimated headspace volume in the plastic bag in the assay was approximately 500–600 mL. The bags were incubated at ~25 °C in a well-lit space. Disease incidence was recorded on days 2, 6, and 8 post-inoculation.

### 2.9. Statistical Analysis

All statistical analyses were performed in R (v4.2.2; R Core Team, R Foundation for Statistical Computing, Vienna, Austria). Differences in VOC concentrations among treatments were assessed using the Kruskal–Wallis test. When significant (*p* < 0.05), pairwise comparisons were performed using Dunn’s test with Holm correction. Effect sizes (epsilon-squared, ε^2^) were calculated to aid interpretation.

For the grape berry in vivo assay, infection incidence was compared using the Mann–Whitney U test with Holm adjustment, and effect sizes were expressed as Cliff’s delta (δ). Disease progression was summarised using the area under the disease progress curve (AUDPC).

EC_50_ values were estimated with the drc package using LL.4 or CRS.4c models, as described in Section 2.7.

## 3. Results and Discussion

### 3.1. HS-SPME-GC/MS Determination of VOCs in Grape and Fermentation Culture Conditions

Chemical screening methods are essential for identifying and quantifying nature-based biologically active compounds. To this end, two HS-SPME-GC/MS methods were developed, validated, and applied to determine 56 VOCs under fermentation conditions using two grape-based model media. Specifically, SCM medium supplemented with Pinot noir grape skins and GJM (diluted grape juice) were selected because they reflect different grape environments: grape-surface-associated nutrients and grape juice composition.

Since some VOCs are formed during fermentation, yeast cultures were grown under static and shaking conditions to evaluate the effect of mixing, which influences oxygen availability and may enhance VOC loss via aeration. The linearity range for each VOC was optimised to enable their detection and quantification in all samples. Because solid-phase microextraction can be influenced by the sample matrix composition, separate calibration curves for grape juice (GJM) and grape-skin-based medium (SCM) were established.

Small differences in calibration slopes were observed for esters and higher alcohols between GJM and SCM, but larger matrix effects were detected for volatile phenols and for terpenes. For several compounds (e.g., isoamyl acetate, ethyl dodecanoate, 4-vinyl guaiacol, linalool, and vitispiranes), the calibration slopes differed by more than 15% between the media. All samples underwent recovery testing to assess analytical accuracy. The majority of analytes showed recoveries within the acceptable 80–120% range. A few compounds fell outside this interval; however, these analytes were not central to the objectives of the study and their deviations do not affect the interpretation of the results.

LOQ values were sufficiently low to capture the VOCs produced during cultivation, with a repeatability (RSDs) and inter-day precision of approximately 10%. Overall, the methods proved to be robust for profiling yeast-derived VOCs under different grape-derived microenvironment conditions.

### 3.2. VOCs Concentration Ranges Prior to Yeast Cultivation

To evaluate VOC generation by yeast metabolism, the baseline concentrations of all 56 VOCs were determined in uninoculated GJM and SCM media.

**Figure 1:**
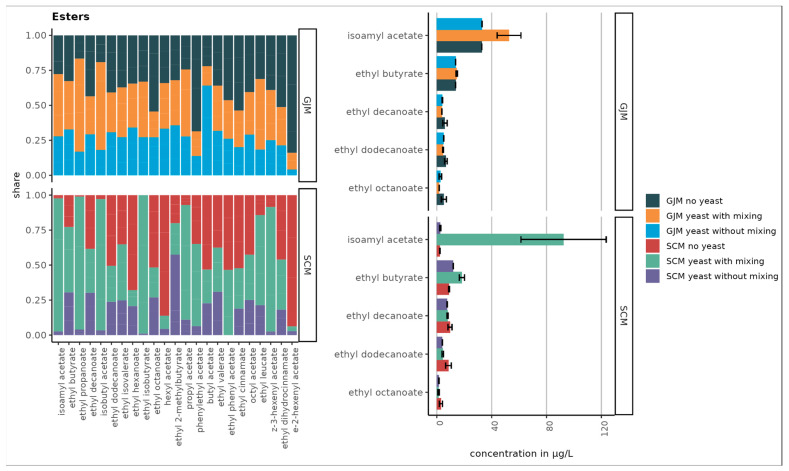
The top five compounds with the highest ester concentrations in SCM and GJM media under different treatments (no yeast, yeast without mixing, and yeast with mixing) are shown in µg/L (right chart). Error bars represent the standard deviations of the means.

**Figure 2:**
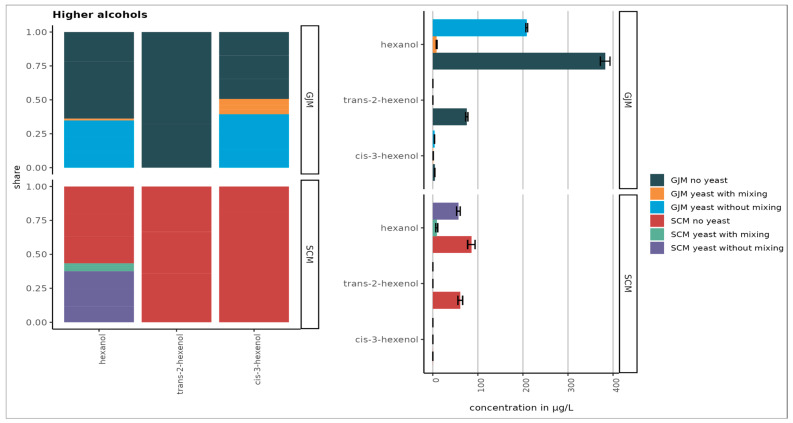
Concentrations of higher alcohols in SCM and GJM treatments (µg/L). Error bars represent the standard deviation of the mean.

**Figure 3:**
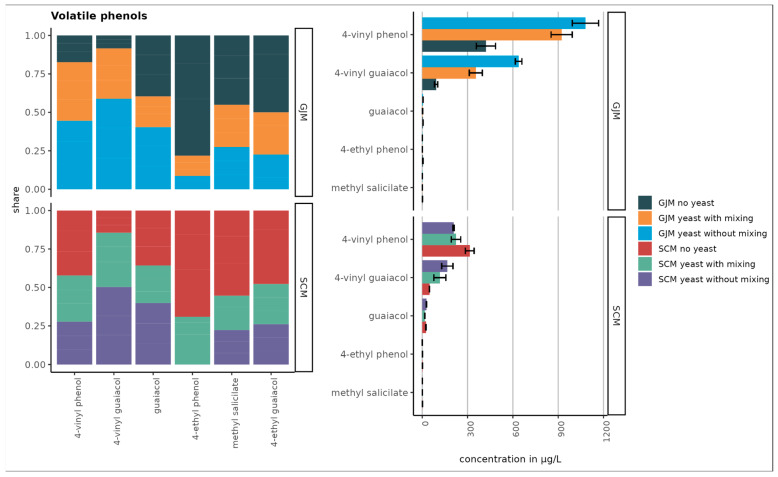
Concentration of volatile phenols in SCM and GJM treatments under different inoculation conditions (µg/L). Error bars represent the standard deviation of the mean.

**Figure 4:**
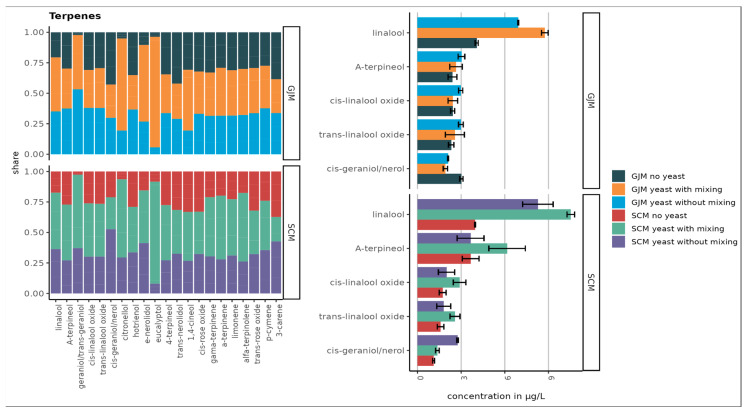
Top 10 terpenes in SCM and GJM media (µg/L). Error bars represent the standard deviation of the mean.

**Figure 5:**
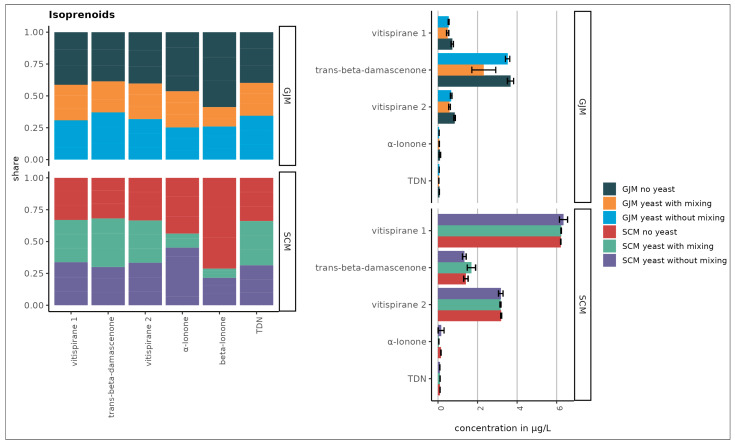
Nor-isoprenoids in SCM and GJM media (µg/L). Error bars represent the standard deviation of the mean.

As shown in Figure 1, Figure 2, Figure 3, Figure 4 and Figure 5, the initial concentrations varied considerably between the two media. For higher alcohols, hexanol and 3-cis-hexenol were >4-fold higher in GJM than in SCM. Among the volatile phenols, 4-vinyl guaiacol, 4-vinyl phenol, and 4-ethyl phenol were more abundant in GJM, whereas 4-ethylguaiacol dominated in SCM. The methyl salicylate levels were similar in both groups.

The esters showed striking differences, with isoamyl acetate being the most abundant ester in GJM, with a 20-fold lower level in SCM. Multiple other esters (e.g., ethyl propanoate, isobutyl acetate, butyl acetate, and hexyl acetate) were >2-fold higher in GJM. In the norisoprenoid group, trans-β-damascenone was elevated in GJM, whereas vitispiranes 1 and 2 were higher in SCM, as shown in Figure 5. For terpenes, only cis-geraniol (nerol) was substantially higher in the GJM.

Overall (Figure 3), volatile phenols were the most abundant class, followed by higher alcohols, esters, terpenes, and norisoprenoids. Among the uninoculated media, hexanol reached the highest concentration (~380 µg/L). These differences between grape juice and grape surface–based models align with previously reported matrix-dependent VOC compositions.

### 3.3. Formation of VOCs During Yeast Growth

Primary grape-derived VOCs (C6 alcohols, esters, terpenes, and norisoprenoids) typically persist throughout fermentation; however, yeast metabolism also contributes significantly to VOC formation via the enzymatic release of glycosidically bound compounds and de novo synthesis.

During cultivation, the VOC profiles shifted markedly. The concentrations of C6 alcohols declined strongly in both GJM and SCM, particularly under shaking (Figure 2). This decrease, especially in hexanol, contrasts with the literature reports for *Saccharomyces cerevisiae* and may reflect species-specific yeast metabolism or evaporation losses during mixing.

In contrast, several VOC groups increased. Volatile phenols, such as 4-vinyl guaiacol, increased in both media, whereas 4-vinyl phenol increased only in SCM, and other phenols decreased. Esters showed the strongest increases, particularly under shaking, with pronounced increases in ethyl propanoate, isoamyl acetate, isobutyl acetate, ethyl butyrate, phenylethyl acetate, and others. However, the treatment effects were not statistically significant (*p* > 0.05).

No major changes occurred in norisoprenoids, although vitispiranes 1 and 2 dominated the group (Figure 5) and were selected for subsequent antifungal assays due to limited prior studies. Higher alcohols, which were depleted rather than synthesised, were excluded from further antifungal testing.

Shaking enhanced aeration, consistent with the literature [47], leading to faster fermentation, higher cell viability, and increased biomass.

### 3.4. Impact of the Shaking on VOCs Production During Yeast Growth

Mixing had distinct effects on the VOC profile. Overall, shaking reduced the higher alcohols in both GJM and SCM, whereas phenol reductions were observed only in GJM.

In total, 23 esters were detected, with Figure 1 and Figure 2 showing the 10 most abundant esters. In GJM, isoamyl acetate levels in “yeast with shaking” were 37% higher than “yeast without shaking” and 38% higher than in uninoculated controls. Ethyl propanoate and isobutyl acetate increased by 70–76% when shaken. Although elevated, the differences in GJM were not statistically significant (*p* > 0.05).

In SCM, the effects were more pronounced: shaking increased ethyl propanoate (97%), isoamyl acetate (91%), and isobutyl acetate (87%) compared to the controls, with statistically significant differences (*p* < 0.05).

In the “no yeast” treatments of both GJM and SCM, we observed lower average concentrations of the higher alcohols. This reduction may result from the evaporation or oxidation of higher alcohols under shaking conditions, leading to the formation of other products [47].

The largest increases in volatile phenol concentrations were observed for 4-vinyl phenol and 4-vinyl guaiacol in the GJM and for 4-vinyl guaiacol in the SCM medium. However, the differences between the treatments were not statistically significant (*p* > 0.05). Trace amounts of volatile phenols are naturally present in grape juice, but the majority are produced by yeast during fermentation. Topić Božič et al. [48] reported that *P. guilliermondii* ZIM624 exhibits high hydroxycinnamate decarboxylase activity, which could explain the decarboxylation of hydroxycinnamic acids and formation of volatile phenols (Figure 3).

A total of 27 terpenes were detected. Figure 4 presents the ten compounds with the highest concentrations. The largest increases were observed for linalool and trans-geraniol in GJM, and for linalool, α-terpineol, trans-geraniol, and citronellol in SCM. However, no statistically significant differences were observed between the treatments (*p* > 0.05).

Overall, terpene concentrations were slightly higher in the SCM samples (Figure 4), likely due to the addition of dried grape skins, which contain higher levels of free and glycosylated monoterpenes compared with pulp or juice. Additionally, *P. guilliermondii* ZIM624 exhibits moderate glycosidase activity, which may hydrolyse terpene glycosides into free volatile terpenes, while acidic grape-based media may further facilitate this release [39]. When comparing the concentration of nor-isoprenoids in samples that were static or mixed during incubation, no major difference was observed between the GJM and SCM media (Figure 5).

Although several VOCs (e.g., isoamyl acetate, ethyl propanoate, and 4-vinyl guaiacol) showed moderate to large effect sizes (ε^2^ ≈ 0.25–0.45), none of the treatment effects remained statistically significant after Holm correction (Table S4), which is consistent with the observed qualitative trends and limited replication (*n* = 3).

### 3.5. Antifungal Activity of Selected Synthetic VOCs Against B. cinerea Growth

**Figure 6:**
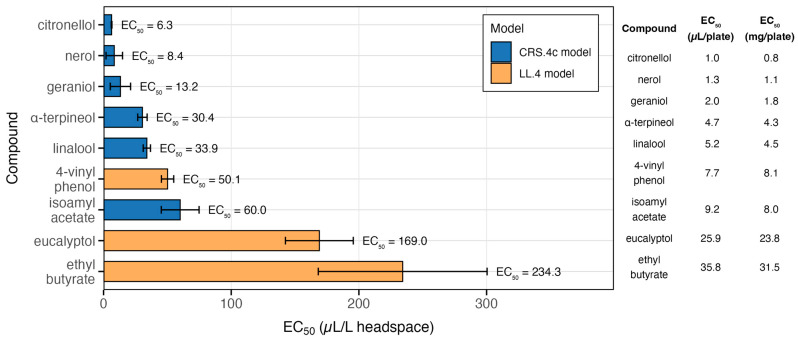
Antifungal activity of selected VOCs against *B. cinerea* F61. The bars represent the EC_50_ values (µL/L airspace) estimated using the best-fitting dose–response model for each compound (constant relative slope model, CRS.4c, or four-parameter log-logistic model, LL.4). The colours indicate the selected model. Error bars represent the 95% confidence intervals. EC_50_ values (µL/L) are shown next to each bar. The table on the right provides the corresponding EC_50_ doses applied per plate (µL/plate and mg/plate), allowing comparison of effective mass across compounds.

A set of 13 VOCs was selected for antifungal testing because they represented the most abundant and consistently yeast-modulated compounds detected in our HS-SPME–GC–MS analyses, covering the dominant chemical classes produced by *P. guilliermondii* ZIM624. Their antifungal activities against *B. cinerea* were quantified using dose–response modelling. EC_50_ values (µL VOC per litre of headspace) were estimated using four-parameter log-logistic (LL.4) or constant relative slope (CRS.4c) models, with model choice based on AIC (Akaike Information Criterion) and parameter stability. The full set of EC_50_ estimates, standard errors, confidence intervals, and slope parameters is provided in Supplementary Table S5, and the most active compounds are visualised in Figure 6.

As in all closed fumigation assays using the widely applied double-sandwich Petri-dish system, some VOC loss due to volatilization and partitioning into agar and polystyrene surfaces is expected. However, because all compounds were tested under identical and commonly used exposure conditions, the nominal headspace dose remains a robust comparative metric for evaluating EC_50_ values across VOCs.

Among the tested VOCs, citronellol (6.3 µL/L), nerol (8.4 µL/L), and geraniol (13.2 µL/L) showed the strongest antifungal activity, with EC_50_ values below 15 µL/L. α-terpineol (30.4 µL/L) and linalool (33.9 µL/L) also exhibited significant inhibitory activity but required higher concentrations, whereas 4-vinyl phenol was moderately effective (50.1 µL/L).

Isoamyl acetate inhibited *B. cinerea* growth at 60 µL/L, while eucalyptol (169 µL/L) and ethyl butyrate (234.3 µL/L) were weak inhibitors. For compounds where neither the LL.4 nor the CRS.4c model provided a satisfactory fit, such as vitispirane, isobutyl acetate, and ethyl propionate, the EC_50_ estimates should be regarded as uncertain, as these compounds showed only limited to weak activity against *B. cinerea*, on the other hand 4-vinyl guaiacol displayed no inhibitory effect within the tested concentration range. The results highlight big differences in antifungal potential among VOC classes. Monoterpene alcohols were the most effective inhibitors of *B. cinerea*, with EC_50_ values below 35 µL/L. These compounds were at least 2–5 times more active than the phenolic derivatives and 5–20 times more active than the esters (Table S5; Figure 6).

Previous reports have shown that oxygenated monoterpenes belong to the “high-potency” group of volatiles in vapour-phase antifungal assays. Both pure compounds and essential oils rich in these compounds (e.g., oregano or thyme oils) [49,50] show strong antifungal activity, which is in line with the potency of our most active compounds, particularly citronellol, geraniol, and nerol. Linalool (33.9 µL/L) and α-terpineol (30.4 µL/L) were slightly less active but remained within a strongly inhibitory range. Their activity has been repeatedly demonstrated in both contact and vapour assays [51]. Several studies suggest that monoterpenes can affect fungal membrane integrity, potentially involving ergosterol-associated processes [51,52], although the precise mechanisms may vary between species. Linalool has been reported to inhibit not only *B. cinerea* [53,54] but also a range of other phytopathogenic fungi and bacteria [52].

Our results extend those of Huang et al. [20], who tested a set of *Candida*-derived esters in the same Petri dish airspace system and reported that compounds such as isoamyl acetate, isobutyl acetate, and ethyl propionate displayed only moderate to weak activity. Our findings are fully consistent with this trend: esters in our fumigation assay required much higher concentrations (60–704 µL/L) to achieve 50% inhibition, placing them among the least effective VOCs (Table S5). Yeast-derived isoamyl acetate, previously noted as an inhibitor of *B. cinerea* growth [55], was again markedly less effective than oxygenated monoterpenes in our assay. Similarly, phenolic derivatives such as 4-vinyl phenol and 4-vinyl guaiacol were weak inhibitors, with the latter not reaching 50% inhibition even at the maximum tested concentration.

Building on these observations, a comparison of the EC_50_ values (mg/plate) presented in Figure 6 for the nine most potent VOCs with their measured concentrations in fermented GJM and SCM samples provides further insight into their potential relevance in real matrices. This comparison shows that not only citronellol and nerol (both with EC_50_ values below 10 µL/L of headspace) have biofumigation potential, but that compounds such as 4-vinylphenol (≈300 µg/L in SCM and up to 1000 µg/L in GJM) and isoamyl acetate (up to 60 µg/L) may also contribute substantially. Although these latter compounds exhibit EC_50_ values roughly three times higher, their concentrations in the fermented samples are far greater than those of the monoterpenols (citronellol < 1 µg/L; nerol ≈ 2 µg/L), suggesting that they may play a more significant role in situ than their intrinsic potencies alone would indicate. Furthermore, because GJM and SCM contain complex mixtures of VOCs, the combined presence of multiple compounds could produce stronger inhibitory effects against *B. cinerea* than any single molecule alone. A potential synergistic or additive interaction among these VOCs, therefore, cannot be excluded.

Accordingly, recent studies have demonstrated that combinations of volatile compounds can be more effective than individual components, indicating additive or synergistic interactions [56]. In future experiments, it will be crucial to complement these single-compound assays with tests of selected mixtures, as combinations of volatile compounds may display additive or synergistic effects that could more accurately reflect the complexity and biological relevance of yeast-derived volatiles in natural environments.

### 3.6. In Vivo Assay of Antifungal VOCs Activity Produced by P. guilliermondii ZIM624 Against B. cinerea F61 in Grape Berries

**Figure 7:**
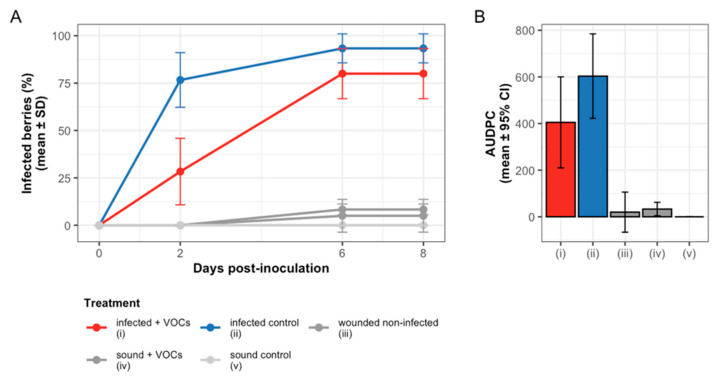
Effect of yeast VOCs on grey mould development in grape berries. (**A**) Infection incidence (% infected berries per bag; mean ± SD, n = 3) for five treatments during 8 days of post-inoculation incubation. (**B**) Area under the disease progress curve (AUDPC; mean ± 95% CI) summarising disease development over time for each treatment. Treatments: (i) infected berries exposed to VOCs from *P. guilliermondii* ZIM624 grown on GJM; (ii) infected berries exposed to sterile GJM (no yeast); (iii) wounded but non-infected berries exposed to yeast VOCs; (iv) sound berries exposed to yeast VOCs; and (v) sound berries exposed to sterile GJM.

The ability of yeast VOCs to inhibit grey mould development was further evaluated using an in vivo grape berry assay with *P. guilliermondii* ZIM624. Throughout the 8-day experiment, VOC exposure consistently suppressed grey mould development in infected berries compared to the infected control (Figure 7A). On day 2, the infection incidence was significantly lower in the VOC treatment (28.3 ± 17.6%) compared with the control (76.7 ± 14.4%). By day 6, infection increased to 80.0 ± 13.2% in VOC-exposed berries but remained below the 93.3 ± 7.6% observed in the control. No further progression was observed between days 6 and 8. As expected, the wounded and sound berries that were not inoculated with *B. cinerea* showed minimal or no infection (Figure 7A, Supplementary Table S6).

To account for disease dynamics over time, AUDPC (Area under the disease progress curve) values were calculated for each replicate (Figure 7B). VOC-treated berries accumulated a mean AUDPC of 405, compared with 603 in the infected control, corresponding to a 32.9% reduction in cumulative disease pressure. Wounded and sound non-infected berries showed very low AUDPC values (≤33), confirming the absence of spontaneous infections. These results demonstrate that VOCs not only delay infection onset but also reduce the overall severity of grey mould development.

Effect size analysis further supported these conclusions. Despite the modest number of biological replicates (*n* = 3 per treatment), VOC exposure produced large Cliff’s δ values on every sampling day (Supplementary Table S6). Effect sizes ranged from −0.67 to −1.00, indicating that infected berries exposed to VOCs almost always exhibited lower infection incidence than the control, even when statistical significance was not reached after Holm correction.

These results demonstrate that *P. guilliermondii* VOCs can suppress *B. cinerea* infection In Vivo by delaying the onset of disease and reducing the overall incidence of grey mould.

Comparable antifungal effects of yeast-derived VOCs have been reported in fruit systems. *C. intermedia* volatiles suppressed grey mould on strawberry [20]. VOCs from *W. anomalus* and *M. pulcherrima* inhibited *B. cinerea* and reduced postharvest disease incidence in strawberries and table grapes [15,57]. Similarly, *Hanseniaspora uvarum* VOCs were shown to lower *B. cinerea* incidence in strawberries and cherries [35].

## 4. Conclusions

Two validated HS-SPME-GC-MS methods were successfully applied to assess VOCs modulated by the biocontrol yeast *P. guilliermondii* ZIM624 in grape juice medium and in synthetic complete medium containing grape skins, simulating two distinct grape-based systems. VOCs were quantified in both media before and six days after yeast activity to evaluate their production during yeast exposure. From these analyses, 56 compounds were identified, of which 13 moderately or abundantly produced VOCs were selected for antifungal testing. Monoterpene alcohols—particularly citronellol, geraniol, and nerol—showed the strongest in vitro inhibitory activity against *B. cinerea*, while In Vivo assays confirmed that the natural VOC blend produced by *P. guilliermondii* ZIM624 can delay infection onset and reduce overall grey mould incidence in grape berries.

Although the observed inhibition was partial, both assays provide consistent evidence that yeast-derived VOCs can contribute to disease suppression in grape-based systems.

From an application standpoint, the active monoterpenes inhibited *B. cinerea* at concentrations (6–15 µL/L) that may be achievable in enclosed storage or packaging environments. Potential delivery modes include slow-release pads, VOC-emitting sachets, or yeast-based biofumigation systems. Future work should evaluate controlled release under commercial storage conditions, aroma transfer to fruit, and regulatory safety limits to determine the feasibility of integrating VOC-based treatments into postharvest management.

## Data Availability

The original contributions presented in the study are included in the article/Supplementary Material; further inquiries can be directed to the corresponding author.

## Supplementary Materials

The following supporting information can be downloaded at: https://www.mdpi.com/article/10.3390/foods15010119/s1, Table S1: Concentration levels of each internal standard in stock solution, mixed stock solution, and final sample solution; Table S2: List of quantified compounds in GJM, including SIM ions, assigned internal standards, calibration ranges and curve parameters, R^2^, recovery, RSD, inter-day precision, LOD, and LOQ.; Table S3: List of quantified compounds with SIM ions, corresponding internal standards, calibration curves, R^2^ values, and recoveries measured in SCM medium. Table S4. Pairwise non-parametric comparison of VOC concentrations in GJM and SCM media. Table S5. Effective concentrations (EC_50_, µL/L airspace) of synthetic volatile organic compounds (VOCs) required to inhibit 50% of *Botrytis cinerea* F61 mycelial growth. Values were estimated using four-parameter log-logistic (LL.4) or constant relative slope (CRS.4c) models, depending on the best model fit. Table S6. Pairwise comparisons of infection incidence between treatments at each sampling day.
