# Supplementary material for: Identification of Volatile Organic Compounds as Natural Antifungal Agents Against Botrytis cinerea in Grape-Based Systems
Source: Foods. 2026 Jan 1;15(1):119. doi: 10.3390/foods15010119 (PMC12785584; doi:10.3390/foods15010119)
Supplement: Supplementary file 1 [file foods-15-00119-s001.zip › Supplementary Table S1_S2_S3_S5.pdf]

## Supplementary materials

**Table S1.** Concentration levels of each internal standard in stock solution, mixed stock solution, and final sample solution.

| *Internal standards* | SIM ion | Stock solution (g/L) | Mixed stock solution (mg/L) | Sample solution (µg/L) |
|---|---|---|---|---|
| **For esters and volatile phenols** | | | | |
| ethyl butyrate-4,4,4 $d_3$ | 74 | 1 | 50 | 25 |
| ethyl-$d_5$ hexanoate | 110 | 1 | 125 | 62.5 |
| ethyl-octanoate $d_{15}$ | 142 | 2.5 | 125 | 62.5 |
| ethyl trans cinnamate $d_5$ | 181 | 2.5 | 0.75 | 37.5 |
| **For higher alcohols** | | | | |
| 2-octanol | 55 | 0.1 | 10 | 50 |
| **For terpenes** | | | | |
| geraniol $d_6$ | 75 | 0.1 | 10 | 50 |
| linalool $d_5$ | 98 | 0.1 | 10 | 50 |
| 2-octanol | 55 | 0.1 | 10 | 50 |
| ethyl trans cinnamate $d_5$ | 181 | 2.5 | 0.75 | 3.75 |
| **For norisoprenoids** | | | | |
| ethyl trans cinnamate $d_5$ | 181 | 2.5 | 0.75 | 3.75 |

**Table S2**: List of quantified compounds in GJM medium, including SIM ions, assigned internal standards, calibration ranges and curve parameters, $R^2$, recovery, RSD, inter-day precision, LOD, and LOQ.

| Compound | Assessed ion (*m/z*) | Calibration range (µg/L) | Calibration curve (y = kx + n) | $R^2$ | Recovery | RSD | Inter-day precision | LOD (µg/L) | LOQ (µg/L) |
|---|---|---|---|---|---|---|---|---|---|
| **Esters** | | | | | | | | | |
| ethyl propanoate[1] | 102 | 1 - 250 | 0.0000770x - 0.0002 | 0.995 | 98.2 | 9.2 | 9.8 | 0.2 | 0.5 |
| ethyl isobutyrate[1] | 116 | 1 - 250 | 0.000149x - 0.0003 | 0.996 | 94.1 | 10.2 | 10.3 | 0.2 | 0.5 |
| propyl acetate[1] | 61 | 1 - 250 | 0.000260x - 0.0003 | 0.996 | 103.0 | 8.6 | 8.9 | 0.2 | 0.5 |
| isobutyl acetate[1] | 56 | 1 - 250 | 0.000400x - 0.0007 | 0.997 | 107.4 | 5.7 | 5.8 | 0.2 | 0.5 |
| ethyl butyrate[1] | 88 | 1 - 250 | 0.000620x - 0.0009 | 0.996 | 100.0 | 7.7 | 7.6 | 0.2 | 0.5 |
| ethyl 2-methylbutyrate[1] | 102 | 1 - 250 | 0.00130x - 0.002 | 0.997 | 98.8 | 8.2 | 8.7 | 0.2 | 0.5 |
| ethyl isovalerate[1] | 88 | 1 - 250 | 0.00146x - 0.006 | 0.998 | 100.5 | 11.2 | 12.1 | 0.2 | 0.5 |
| phenylethyl acetate[7] | 104 | 1 - 250 | 0.227x - 0.03 | 0.994 | 118.9 | 9.5 | 9.9 | 0.2 | 0.5 |

| | | | | | | | | | |
|---|---|---|---|---|---|---|---|---|---|
| butyl acetate[1] | 56 | 1 - 250 | 0.000820 x + 0.0005 | 0.991 | 109.9 | 7.2 | 8.0 | 0.2 | 0.5 |
| isoamyl acetate[1] | 70 | 25 - 5000 | 0.00197x - 0.064 | 0.993 | 92.5 | 5.1 | 5.8 | 0.2 | 0.5 |
| ethyl hexanoate[2] | 88 | 10 - 2500 | 0.000890 x - 0.005 | 0.98 | 104.5 | 7.7 | 8.1 | 0.3 | 1 |
| ethyl octanoate[3] | 88 | 10 - 2500 | 0.00145x - 0.002 | 0.98 | 109.1 | 8.2 | 8.3 | 0.3 | 1 |
| hexyl acetate[2] | 56 | 2.5 - 500 | 0.000910 x - 0.003 | 0.994 | 92.7 | 10.4 | 11.1 | 0.3 | 1 |
| ethyl decanoate[3] | 88 | 2.5 - 500 | 0.00212x - 0.006 | 0.997 | 105.9 | 11.4 | 11.2 | 0.3 | 1 |
| ethyl leucate[2] | 87 | 2.5 - 500 | 0.00009x | 0.999 | 112.0 | 8.8 | 9.2 | 0.3 | 1 |
| ethyl valerate[1] | 88 | 0.25 - 50 | 0.00270x - 0.001 | 0.997 | 93.5 | 7.2 | 7.5 | 0.03 | 0.1 |
| Z-3-hexenyl acetate[2] | 82 | 0.25 - 50 | 0.000910 x - 0.0001 | 0.996 | 112.0 | 9.7 | 10.2 | 0.03 | 0.1 |
| E-2-hexenyl acetate[2] | 82 | 0.25 - 50 | 0.000629 x | 0.997 | 94.0 | 9.2 | 9.6 | 0.03 | 0.1 |
| octyl acetate[3] | 70 | 0.25 - 50 | 0.000672 x - 0.0002 | 0.992 | 92.1 | 7.2 | 7.9 | 0.03 | 0.1 |
| ethyl dodecanoate[3] | 88 | 0.25 - 50 | 0.000580 x - 0.002 | 0.993 | 113.3 | 9.6 | 9.8 | 0.03 | 0.1 |
| ethyl phenyl acetate[7] | 91 | 0.25 - 50 | 0.395x - 0.092 | 0.994 | 103.4 | 6.9 | 7.0 | 0.03 | 0.1 |

| | | | | | | | | | |
|---|---|---|---|---|---|---|---|---|---|
| ethyl cinnamate[7] | 176 | 0.025 – 5 | 0.0536x - 0.007 | 0.997 | 139.1 | 12.6 | 13.2 | 0,005 | 0,01 |
| ethyl dihydro cinnamate[7] | 178 | 0.025 – 5 | 0.122x - 0.003 | 0.9995 | 121.1 | 11.7 | 12.0 | 0,005 | 0,01 |
| **Higher alcohols** | | | | | | | | | |
| Hexanol[6] | 56 | 100 - 20000 | 0.000106 x + 0.009 | 0.98 | 119.9 | 10.5 | 10.4 | 0.3 | 1 |
| Z-3-hexenol[6] | 82 | 2.5 - 500 | 0.000025 x | 0.996 | 119.2 | 8.2 | 8.7 | 0.3 | 1 |
| E-2-hexenol[6] | 82 | 2.5 - 500 | 0.000016 x + 0.0003 | 0.995 | 135.6 | 7.9 | 8.0 | 0.3 | 1 |
| E-3-hexenol[6] | 82 | 2.5 - 500 | 0.000028 x | 0.995 | 120.2 | 9.3 | 10.1 | 0.3 | 1 |
| **Volatile phenols** | | | | | | | | | |
| 4-ethyl phenol[7] | 107 | 5 - 1000 | 0.0432x - 0.195 | 0.998 | 113.2 | 6.6 | 6.7 | 0.1 | 0.3 |
| 4-ethyl guaiacol[7] | 137 | 2.5 - 500 | 0.0773x - 0.020 | 0.9997 | 119.5 | 8.5 | 8.6 | 0.1 | 0.3 |
| methyl salicilate[7] | 120 | 0.5 - 100 | 0.105x - 0.049 | 0.998 | 122.9 | 11.2 | 11.9 | 0.1 | 0.3 |
| 4-vinyl guaiacol[7] | 150 | 5 - 1000 | 0.00340x - 0.058 | 0.996 | 106.6 | 7.9 | 8.4 | 0.1 | 0.3 |
| 4-vinyl phenol[7] | 120 | 25 - 5000 | 0.000580 x - 0.0610 | 0.995 | 118.8 | 8.4 | 8.3 | 0.1 | 0.3 |
| Guaiacol[7] | 124 | 1 - 250 | 0.0133x - 0.0066 | 0.9993 | 105.6 | 9.1 | 9.4 | 0.1 | 0.3 |
| **Terpenes** | | | | | | | | | |

| | | | | | | | | | |
|---|---|---|---|---|---|---|---|---|---|
| 3-carene[6] | 93 | 0.01 - 2 | 0.00492x | 0.998 | 77.2 | 12.2 | 12.9 | 0.002 | 0.005 |
| α-terpinene[6] | 136 | 0.01 - 2 | 0.00241x | 0.998 | 88.1 | 8.7 | 9.0 | 0.001 | 0.004 |
| 1,4-cineole[6] | 111 | 0.01 - 2 | 0.00382x | 0.998 | 105.1 | 9.3 | 9.2 | 0.002 | 0.005 |
| l imonene[6] | 136 | 0.01 - 2 | 0.00449x - 0.0001 | 0.998 | 85.7 | 5.7 | 6.0 | 0.001 | 0.004 |
| eucalyptol[6] | 108 | 0.01 - 2 | 0.00147x | 0.997 | 110.6 | 6.1 | 6.5 | 0.002 | 0.005 |
| γ-terpinene[6] | 136 | 0.01 - 2 | 0.00128x | 0.998 | 84.5 | 8.1 | 8.9 | 0.002 | 0.005 |
| p-cymene[6] | 119 | 0.01 - 2 | 0.0194x - 0.0001 | 0.9999 | 85.2 | 8.6 | 8.7 | 0.002 | 0.005 |
| α-terpinolene[6] | 136 | 0.01 - 2 | 0.00512x - 0.0001 | 0.997 | 86.2 | 5.3 | 5.8 | 0.001 | 0.004 |
| cis-rose oxide[6] | 139 | 0.05 - 10 | 0.00800x - 0.0009 | 0.9999 | 88.3 | 7.7 | 8.1 | 0.002 | 0.005 |
| trans-rose oxide[6] | 139 | 0.05 - 10 | 0.00290x - 0.0001 | 0.9998 | 84.3 | 9.2 | 10.0 | 0.003 | 0.008 |
| trans-linalool oxide[5] | 94 | 0.5 - 100 | 0.000631x - 0.0007 | 0.994 | 92.6 | 10.1 | 10.5 | 0.003 | 0.009 |
| cis-linalool oxide[5] | 94 | 0.5 - 100 | 0.000383x - 0.0005 | 0.994 | 90.6 | 11.2 | 12.0 | 0.003 | 0.009 |
| vitispirane 1[7] | 192 | 0.5 - 100 | 0.0360x - 0.015 | 0.996 | 95.3 | 9.9 | 9.8 | 0.002 | 0.006 |
| vitispirane 2[7] | 192 | 0.5 - 100 | 0.0622x - 0.031 | 0.995 | 91.8 | 10.6 | 10.7 | 0.002 | 0.006 |
| Linalool[5] | 93 | 0.5 - 100 | 0.00550x - 0.010 | 0.993 | 86.0 | 6.7 | 6.8 | 0.003 | 0.008 |

| | | | | | | | | | |
|---|---|---|---|---|---|---|---|---|---|
| 4-terpineol[5] | 111 | 0.05 - 10 | 0.00572x - 0.001 | 0.996 | 85.3 | 6.3 | 6.9 | 0.002 | 0.005 |
| Hotrienol[5] | 71 | 0.5 - 100 | 0.0128x - 0.010 | 0.9991 | 87.3 | 9.6 | 10.5 | 0.003 | 0.009 |
| α -terpineol[5] | 93 | 0.5 - 100 | 0.00319x - 0.002 | 0.998 | 90.6 | 5.4 | 6.1 | 0.002 | 0.006 |
| TDN[7] | 157 | 0.05 - 10 | 0.772x - 0.030 | 0.9992 | 77.6 | 8.3 | 8.9 | 0.002 | 0.006 |
| cis-geraniol (nerol)[4] | 93 | 0.5 - 100 | 0.00180x - 0.003 | 0.9998 | 82.1 | 9.4 | 9.3 | 0.003 | 0.01 |
| trans- β -damascenone[7] | 190 | 0.05 - 10 | 0.0862x - 0.037 | 0.998 | 83.8 | 10.7 | 11.2 | 0.002 | 0.006 |
| trans geraniol [4] | 136 | 0.5 - 100 | 0.000117 x + 0.0001 | 0.9991 | 96.1 | 9.5 | 10.0 | 0.003 | 0.01 |
| α -ionone[7] | 121 | 0.01 - 2 | 0.318x - 0.0006 | 0.996 | 74.6 | 8.5 | 8.5 | 0.003 | 0.01 |
| β -ionone[7] | 177 | 0.01 - 2 | 0.476x + 0.0003 | 0.9995 | 92.3 | 7.9 | 8.0 | 0.003 | 0.01 |
| trans-nerolidol[4] | 93 | 0.05 - 10 | 0.00110x - 0.0002 | 0.999 | 77.6 | 11.4 | 11.6 | 0.003 | 0.009 |
| E-nerolidol[4] | 93 | 0.05- 10 | 0.00147x - 0.0002 | 0.9993 | 78.0 | 10.2 | 11.1 | 0.003 | 0.009 |
| citronellol [4] | 95 | 0.5 - 100 | 0.00210x + 0.0009 | 0.9992 | 97.7 | 6.8 | 7.0 | 0.002 | 0.006 |

[1] ethyl butyrate-4,4,4 $d_3$

[2]ethyl-$d_5$ hexanoate

[3]ethyl-octanoate $d_{15}$

[4]geraniol $d_6$

[5]linalool $d_5$

[6]2-octanol

[7]ethyl trans cinnamate $d_5$

**Table S3: List of quantified compounds with SIM ions, corresponding internal standards, calibration curves, $R^2$ values, and recoveries measured in SCM medium.**

| [a]Compound | Calibration curve (y = kx +n) | $R^2$ | Recovery |
|---|---|---|---|
| **Esters** | | | |
| ethyl propanoate[1] | 0.000078x + 0.000084 | 0.9998 | 105.3 |
| ethyl isobutyrate[1] | 0.000158x - 0.000182 | 0.9991 | 91.4 |
| propyl acetate[1] | 0.00026x + 0.00032 | 0.9997 | 108.7 |
| isobutyl acetate[1] | 0.00045x - 0.00014 | 0.9997 | 101.3 |
| ethyl butyrate[1] | 0.00065x + 0.00023 | 0.9998 | 103.9 |
| ethyl 2-methylbutyrate[1] | 0.0014x - 0.0014 | 0.9994 | 95.5 |
| ethyl isovalerate[1] | 0.001612x - 0.002495 | 0.9997 | 95.2 |
| phenylethyl acetate[7] | 0.2447x + 0.0019 | 0.9998 | 123.7 |
| butyl acetate[1] | 0.000921x - 0.000241 | 0.999 | 103.6 |
| isoamyl acetate[1] | 0.00158x + 0.096932 | 0.998 | 123.6 |
| ethyl hexanoate[2] | 0.00094x + 0.0082 | 0.997 | 102.2 |
| ethyl octanoate[3] | 0.001413x + 0.024421 | 0.995 | 107.7 |
| hexyl acetate[2] | 0.00085x + 0.00082 | 0.9997 | 103.6 |
| ethyl decanoate[3] | 0.00233x - 0.01626 | 0.997 | 98.8 |
| ethyl leucate[2] | 0.00009x + 0.0004 | 0.994 | 116.9 |
| ethyl valerate[1] | 0.0027x - 0.0005 | 0.9998 | 98.2 |
| Z-3-hexenyl acetate[2] | 0.00102x + 0.00012 | 0.9994 | 105.7 |
| E-2-hexenyl acetate[2] | 0.000543x + 0.000266 | 0.998 | 109.3 |
| octyl acetate[2] | 0.00065x - 0.000058 | 0.9993 | 93.4 |
| ethyl dodecanoate[3] | 0.00027x - 0.00077 | 0.997 | 122.4 |
| ethylphenyl acetate[7] | 0.3953x + 0.0176 | 0.9999 | 116.2 |
| ethyl cinnamate[7] | 0.0523x - 0.0084 | 0.9992 | 146.1 |
| ethyl dihydrocinnamate[7] | 0.11995x - 0.00339 | 0.998 | 135.1 |
| **Higher alcohols** | | | |
| hexanol [6] | 0.000117x + 0.016524 | 0.98 | 116.3 |

| | | | |
|---|---|---|---|
| Z-3-hexenol[6] | 0.000026x + 0.000072 | 0.997 | 117.4 |
| E-2-hexenol[6] | 0.000016x + 0.000214 | 0.9998 | 128.2 |
| E-3-hexenol[6] | 0.000027x + 0.000105 | 0.998 | 129.9 |
| **Volatile phenols** | | | |
| 4-ethyl phenol[7] | 0.0444x + 0.0075 | 0.9998 | 120.2 |
| 4-ethyl guaiacol[7] | 0.0823x - 0.0735 | 0.9999 | 120.5 |
| methyl salicilate[7] | 0.114096x - 0.0821 | 0.99993 | 122.9 |
| 4-vinyl guaiacol[7] | 0.0012x - 0.0281 | 0.998 | 116.5 |
| 4-vinyl phenol[7] | 0.00048x - 0.06697 | 0.998 | 115.5 |
| Guaiacol[7] | 0.0135x - 0.0089 | 0.9998 | 112.4 |
| **Terpenes** | | | |
| 3-carene[6] | 0.0035x - 0.00003 | 0.998 | 80.5 |
| α -terpinene[6] | 0.001646x + 0.0000007 | 0.99991 | 94.7 |
| 1,4-cineole[6] | 0.003933x - 0.00006 | 0.998 | 99.3 |
| limonene [6] | 0.003353x - 0.000002 | 0.99997 | 95.0 |
| eucalyptol [6] | 0.00144x + 0.00001 | 0.999 | 102.9 |
| γ -terpinene[6] | 0.000909x + 0.000005 | 0.9995 | 90.1 |
| p-cymene[6] | 0.0162x - 0.00004 | 0.996 | 85.9 |
| α -terpinolene[6] | 0.003554x - 0.000014 | 0.99994 | 93.3 |
| cis-rose oxide[6] | 0.0076x - 0.0005 | 0.9996 | 86.9 |
| trans-rose oxide[6] | 0.0027x - 0.0002 | 0.9997 | 86.3 |
| trans-linalool oxide[5] | 0.000528x + 0 | 0.996 | 102.0 |
| cis-linalool oxide[5] | 0.000312x + 0 | 0.994 | 101.8 |
| vitispirane 1[7] | 0.097148x - 0.598 | 0.996 | 55.9 |
| vitispirane 2[7] | 0.1019x - 0.2958 | 0.998 | 59.2 |
| linalool[5] | 0.0044x - 0.0037 | 0.98 | 102.5 |
| 4-terpineol[5] | 0.00486x - 0.00006 | 0.994 | 101.0 |
| hotrienol[5] | 0.0102x - 0.0037 | 0.998 | 86.8 |
| α -terpineol[5] | 0.00255x + 0.00146 | 0.994 | 113.5 |
| TDN[7] | 0.8277x - 0.0724 | 0.998 | 77.3 |
| cis-geraniol (nerol)[4] | 0.00097x - 0.00039 | 0.9998 | 81.0 |
| trans-β-damascenone[7] | 0.0849x + 0.0199 | 0.992 | 88.9 |
| trans geraniol[4] | 0.000102x + 0.000149 | 0.9994 | 106.5 |
| α -ionone[7] | 0.2847x - 0.0076 | 0.998 | 81.1 |
| β-ionone[7] | 0.4626x - 0.0033 | 0.999 | 90.9 |
| trans-nerolidol[4] | 0.00097x - 0.00034 | 0.995 | 71.5 |
| E-nerolidol[4] | 0.00122x - 0.00042 | 0.997 | 77.1 |

| | | | |
|---|---|---|---|
| citronellol[4] | 0.00202x - 0.00028 | 0.99998 | 90.5 |

[a]Assessed ions, Calibration ranges, RSDs, LODs and LOQs are already presented in Table S2.

[1]ethyl butyrate-4,4,4 $d_3$

[2]ethyl-$d_5$ hexanoate

[3]ethyl-octanoate $d_{15}$

[4]geraniol $d_6$

[5]linalool $d_5$

[6]2-octanol

[7]ethyl trans cinnamate $d_5$

.

**Table S5.** Effective concentrations ($EC_{50}$, µL/L airspace) of synthetic volatile organic compounds (VOCs) required to inhibit 50% of *Botrytis cinerea* F61 mycelial growth. Values were estimated using four-parameter log-logistic (LL.4) or constant relative slope (CRS.4c) models, depending on best model fit.

| Compound | Model[1] tested | $EC_{50}$ (mean ± SE[2]) (µL/L) | 95% CI[3] (lower-upper) | Hill slope ± SE[2] | AIC[4] | Summary of model fit assessment |
|---|---|---|---|---|---|---|
| citronellol | LL.4 | 6.7 ± 0.1 | 6.5–6.9 | 3.99 ± 0.34 | 94 | Best model: CRS.4c (lower AIC) |
| | CRS.4c | 6.3 ± 0.1 | 6.0–6.7 | 5.72 ± 1.91 | 89 | |
| geraniol | LL.4 | 15.8 ± 0.5 | 14.7–16.9 | 3.39 ± 0.47 | 112 | Best model: CRS.4c (lower AIC); $EC_{50}$ unstable (wide CI) |
| | CRS.4c | 13.2 ± 3.8 | 5.3–21.2 | 8.93 ± 27.86 | 97 | |
| nerol | LL.4 | 14.7 ± 0.8 | 13.1–16.4 | 2.63 ± 0.34 | 129 | Best model: CRS.4c (lower AIC); $EC_{50}$ unstable (wide CI) |
| | CRS.4c | 8.4 ± 3.1 | 1.9–14.9 | 5.20 ± 1.91 | 116 | |
| $\alpha$-terpineol | LL.4 | 31.2 ± 1.0 | 29.2–33.3 | 3.65 ± 0.41 | 109 | Best model: CRS.4c (lower AIC) |
| | CRS.4c | 30.4 ± 1.7 | 26.7–34.1 | 5.42 ± 1.87 | 104 | |
| linalool | LL.4 | 36.3 ± 0.9 | 34.3–38.3 | 3.44 ± 0.41 | 107 | Best model: CRS.4c (lower AIC) |
| | CRS.4c | 33.9 ± 1.3 | 31.1–36.8 | 4.10 ± 0.52 | 104 | |
| 4-vinyl phenol | LL.4 | 50.1 ± 2.3 | 45.3–55.0 | 3.41 ± 0.40 | 131 | Models equivalent; LL.4 selected (more stable) |
| | CRS.4c | 48.4 ± 3.5 | 41.0–55.8 | 3.55 ± 0.41 | 131 | |
| isoamyl acetate | LL.4 | 63.6 ± 7.3 | 48.1–79.1 | 3.36 ± 1.78 | 176 | Models equivalent; CRS.4c selected (more stable). |
| | CRS.4c | 60.0 ± 7.0 | 45.2–74.8 | 4.65 ± 5.11 | 176 | |
| vitispirane[5] | LL.4 | 165.7 ± 106.5 | -59.0–390.3 | 2.07 ± 1.92 | 109 | LL.4: $EC_{50}$ not reached within tested range; no model convergence; CRS.4c: no model convergence, $EC_{50}$ not estimable |
| | CRS.4c | > max tested conc. | - | - | - | |
| isobutyl acetate[5] | LL.4 | 196.9 ± 53.2 | 84.6–309.2 | 4.56 ± 4.18 | 119 | Best model: CRS.4c (lower AIC); LL.4 unstable |
| | CRS.4c | 162.6 ± 39.9 | 78.4–246.9 | 12.09 ± 29.50 | 114 | |
| eucalyptol | LL.4 | 169.0 ± 12.6 | 142.4–195.6 | 1.57 ± 0.15 | 100 | Best model: LL.4 (lower AIC) |
| | CRS.4c | 131.8 ± 8.8 | 113.3–150.3 | 2.15 ± 0.23 | 108 | |
| ethyl butyrate | LL.4 | 234.3 ± 31.3 | 168.2–300.4 | 1.66 ± 0.23 | 114 | Best model: LL.4 (lower AIC) |
| | CRS.4c | 162.7 ± 11.6 | 138.2–187.2 | 2.72 ± 0.45 | 117 | |
| ethyl propionate[5] | LL.4 | | | | 123 | |
| | | 466.3 ± 1028.2 | -1702.9–2635.6 | 2.39 ± 3.01 | | Best model: CRS.4c; LL.4 $EC_{50}$ unstable |
| | CRS.4c | 663.7 ± 47.7 | 563.0–764.4 | 2.10 ± 0.60 | 123 | |
| 4-vinyl guaiacol[5] | LL.4 | 703.6 ± 2225.0 | -3990.8–5398.0 | -1.48 ± 3.60 | 113 | LL.4: $EC_{50}$ not reached within tested dose range; CRS.4c: no model convergence, $EC_{50}$ not estimable |
| | CRS.4c | > max tested conc. | - | - | - | |

[1] Open source package “drc” in R. LL.4 is four-parameter log-logistic model, and CRS.4c is the constant relative slope 4-parameter logistic model from the drc package.

[2] SE = standard error.

[3] CI = 95% confidence interval for $EC_{50}$

[4]AIC = Akaike Information Criterion

[5] For these compounds, 50% inhibition of *B. cinerea* growth was not achieved within the tested concentration range (max tested: 735 μL/L). $EC_{50}$ values are therefore extrapolated by the model or expressed as “> max tested concentration.”
